# Supplementary figures and images for: SARS-CoV-2 mutations among minks show reduced lethality and infectivity to humans
Source: PLoS One. 2021 May 26;16(5):e0247626. doi: 10.1371/journal.pone.0247626 (PMC8153470; doi:10.1371/journal.pone.0247626)

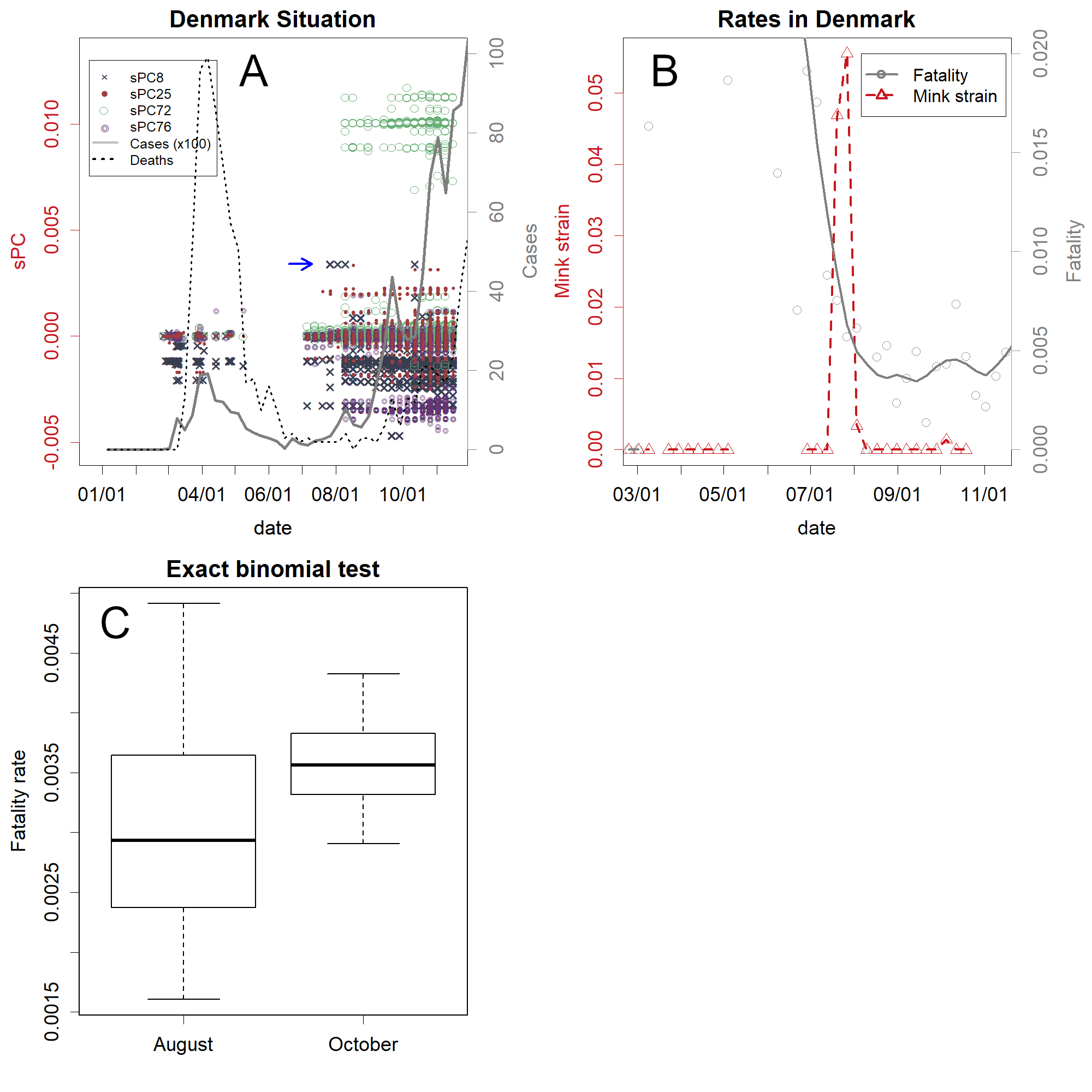

Supplement: S1 Fig — A. Number of confirmed cases, deaths, and PCs for samples. The blue arrow indicates the mink-derived human-virus (sPC8), which are far fewer than those in the Netherlands. B. Fatality rate (the number of deaths in the following week/the number of cases, grey) and percentage of mink-derived human-virus (red). The fatality rate remained fairly constant after the first wave subsided. C. The estimated confidence intervals confirmed the constancy of fatality. (PNG) [file pone.0247626.s001.png]
