# Supplementary material for: SARS-CoV-2 mutations among minks show reduced lethality and infectivity to humans
Source: PLoS One. 2021 May 26;16(5):e0247626. doi: 10.1371/journal.pone.0247626 (PMC8153470; doi:10.1371/journal.pone.0247626)
Supplement: S1 Table — The differences between each variant in 1000 amino acid residues (positions are shown in Fig 1B and 1C by the IDs) and NB-EMC-35-3 | EPI_ISL_577774 (a mink-virus considered to be the same as that of humans). The rates of missense mutations are also shown. *Humans: differences between the mink-derived variants and similar human-virus in group 2, the Netherlands. The mink-derived human-virus were NB-EMC-45-4, NB-EMC-39-5, NB-EMC-7-2, NB-EMC-26-1, NB-EMC-45-3, NB-EMC-39-3, and NB-EMC-41-4. The related variants were NB-EMC-312, ZH-EMC-379, ZH-EMC-844, and ZH-EMC-845. Differences between averages were used. (PDF) [file pone.0247626.s002.pdf]

We gratefully acknowledge the following Authors from the Originating laboratories responsible for obtaining the specimens, as well as the Submitting laboratories where the genome data were generated and shared via GISAID, on which this research is based.

All Submitters of data may be contacted directly via www.gisaid.org

Accession ID	Originating Laboratory	Submitting Laboratory	Authors
EPI_ISL_522992, EPI_ISL_522994, EPI_ISL_522995, EPI_ISL_522996, EPI_ISL_522997, EPI_ISL_522998, EPI_ISL_522999, EPI_ISL_523000, EPI_ISL_523001, EPI_ISL_523002, EPI_ISL_523003, EPI_ISL_523004, EPI_ISL_523005, EPI_ISL_523006, EPI_ISL_523007, EPI_ISL_523008, EPI_ISL_523009, EPI_ISL_523010, EPI_ISL_523014, EPI_ISL_523015, EPI_ISL_523016, EPI_ISL_523017, EPI_ISL_523018, EPI_ISL_523019, EPI_ISL_523020, EPI_ISL_523028, EPI_ISL_523034, EPI_ISL_523035, EPI_ISL_523036, EPI_ISL_523037, EPI_ISL_523038, EPI_ISL_523039, EPI_ISL_523046, EPI_ISL_523053, EPI_ISL_523054, EPI_ISL_523055, EPI_ISL_523056, EPI_ISL_523057, EPI_ISL_523058, EPI_ISL_523059, EPI_ISL_523060, EPI_ISL_523061, EPI_ISL_523074, EPI_ISL_523076, EPI_ISL_523077, EPI_ISL_523079, EPI_ISL_523080, EPI_ISL_523081, EPI_ISL_523082, EPI_ISL_523083, EPI_ISL_523086, EPI_ISL_523087, EPI_ISL_523091, EPI_ISL_523092, EPI_ISL_523093, EPI_ISL_523094, EPI_ISL_523095, EPI_ISL_523096, EPI_ISL_523098, EPI_ISL_523099, EPI_ISL_523101, EPI_ISL_523103, EPI_ISL_523104, EPI_ISL_523105, EPI_ISL_523106, EPI_ISL_523108, EPI_ISL_523109, EPI_ISL_523110, EPI_ISL_523111, EPI_ISL_523112, EPI_ISL_523113, EPI_ISL_523114, EPI_ISL_523117, EPI_ISL_523118, EPI_ISL_523119, EPI_ISL_577755, EPI_ISL_577756, EPI_ISL_577758, EPI_ISL_577768, EPI_ISL_577770, EPI_ISL_577771, EPI_ISL_577783, EPI_ISL_577784, EPI_ISL_577785, EPI_ISL_577786, EPI_ISL_577791, EPI_ISL_577802, EPI_ISL_577803			
see above	Dutch COVID-19 response team	Erasmus Medical Center	OH consortium
EPI_ISL_626340, EPI_ISL_626341, EPI_ISL_626342, EPI_ISL_626343, EPI_ISL_626344, EPI_ISL_626345, EPI_ISL_626346, EPI_ISL_626347, EPI_ISL_626348, EPI_ISL_626349, EPI_ISL_626350, EPI_ISL_626351			
see above	Statens Serum Institute	Statens Serum Institute	Hammer, A.S., Quaade, M.L., Rasmussen, T.B., Fonager, J., Rasmussen, M., Mundbjerg, K., Lohse, L., Strandbygaard, B., Jorgensen, C.S., Afaro-Nunez, A., Rosenstierne, M.W., Halasa, T., Foomsgaard, A., Belsham, G.J. and Botner, A.
EPI_ISL_641414, EPI_ISL_641419, EPI_ISL_641420	Department of Virus and Microbiological Special Diagnostics, Statens Serum Institut, Copenhagen, Denmark	Albertsen lab, Department of Chemistry and Bioscience, Aalborg University, Denmark	Thomas Bruun Rasmussen, Jannik Fonager, Morten Rasmussen
