# Supplementary material for: SARS-CoV-2 mutations among minks show reduced lethality and infectivity to humans
Source: PLoS One. 2021 May 26;16(5):e0247626. doi: 10.1371/journal.pone.0247626 (PMC8153470; doi:10.1371/journal.pone.0247626)
Supplement: Attachment — Submitted filename: Response to Reviewers.docx [file pone.0247626.s003.docx]

5. Review Comments to the Author

Please use the space provided to explain your answers to the questions above. You may also include additional comments for the author, including concerns about dual publication, research ethics, or publication ethics. (Please upload your review as an attachment if it exceeds 20,000 characters)

Thank you for reviewing our manuscript. I have formatted it according to the style required by PLOS ONE, and have revised it considering the comments and questions.

Reviewer #1: The manuscript is well-written. The authors use prinicipal component analysis and show that the evolution of human- and mink SARS-CoV-2 differs completely. In addition real-life data from the Netherlands and Denmark indicate that mink variants are less lethal and infective.

The authors suggest that mink-variants may be used for development of vaccines and therfore advise against culling of mink.

The findings in this paper is very important, but also controversial and political hot stuff. In Denmark it is still debated whether or not the culling of 17 millions mink was the right decision.

I appreciate this comment. I tried to contact Danish officials. However, mink farming was banned in the country and the mink population was culled. The variants that could have supplemented the deficient vaccine were destroyed.

Reviewer #2: This is a nice and informative study. I have few comments and I request the author to clarify the comments.

1) It's not clear how the author derived the following results "Some mink-derived variants infected humans, which accounted for 40% of the total SARS-CoV-2 cases in the Netherlands". The author presented this result in the "Introduction" of the main manuscript and then presented these as a part of results in the Abstract. Reading the article -- I didn't understand how the author derived the figure. Please clarify this.

I rewrote the paragraph adding explanations concerning Fig. 1C, especially for the group indicated by the circle in the figure. I hope this improves the readability of the passage.

2) In method the author mentioned "The axes were identified using 103 mink-virus and 6092 human-virus that were

proportionally selected from each continent. These data may be comparable to 130

million animals; since the mink population is estimated to be 50 million... ,".

How 103 minus 6092 human were comparable to 130 million animals? What does 130 million animals refers?

I regret this shortage. I added an explanation by using an equation.

3) The author used abbreviation of worlds which need elaborated especially in figure legends and methods which now stands alone.

I appreciate the comment. The revised version includes the Materials and Methods section. A summary of all the abbreviations is included. Finally, I tried to find a replacement for DECIPHER, but it seems that this is rather a unique noun.

I hope that these changes meet the requirements and improve the readability of the manuscript. The manuscript was edited by Editage (www.editage.com) for English language.
